# Women's childhood and adult adverse experiences, mental health, and binge drinking: The California Women's Health Survey

**DOI:** 10.1186/1747-597X-3-15

**Published:** 2008-06-06

**Authors:** Christine Timko, Anne Sutkowi, Joanne Pavao, Rachel Kimerling

**Affiliations:** 1 Center for Health Care Evaluation, Department of Veterans Affairs (VA) Health Care System, 795 Willow Road, Menlo Park, CA 94025, USA; 2 Department of Psychiatry and Behavioral Sciences, Stanford University, Stanford, CA 94305, USA; 3 National Center for PTSD, VA Health Care System, 795 Willow Road, Menlo Park, CA 94025, USA

## Abstract

**Background:**

This study examined sociodemographic, physical and mental health, and adult and childhood adverse experiences associated with binge drinking in a representative sample of women in the State of California.

**Materials and methods:**

Data were from the 2003 to 2004 (response rates of 72% and 74%, respectively) California Women's Health Survey (CWHS), a population-based, random-digit-dial annual probability survey sponsored by the California Department of Health Services. The sample was 6,942 women aged 18 years or older.

**Results:**

The prevalence of binge drinking was 9.3%. Poor physical health, and poorer mental health (i.e., symptoms of PTSD, anxiety, and depression, feeling overwhelmed by stress), were associated with binge drinking when demographics were controlled, as were adverse experiences in adulthood (intimate partner violence, having been physically or sexually assaulted, or having experienced the death of someone close) and in childhood (living with someone abusing substances or mentally ill, or with a mother vicimized by violence, or having been physically or sexually assaulted). When adult mental health and adverse experiences were also controlled, having lived as a child with someone who abused substances or was mentally ill was associated with binge drinking. Associations between childhood adverse experiences and binge drinking could not be explained by women's poorer mental health status in adulthood.

**Conclusion:**

Identifying characteristics of women who engage in binge drinking is a key step in prevention and intervention efforts. Binge drinking programs should consider comprehensive approaches that address women's mental health symptoms as well as circumstances in the childhood home.

## Background

Binge drinking has been linked to a broad range of health and psychosocial problems. Although definitions of binge drinking vary somewhat among studies, a commonly used definition for women and men is that of the Centers for Disease Control and Prevention's (CDC) Behavioral Risk Factor Surveillance System: five drinks per drinking occasion at least once during the past 30 days. Rates of binge drinking are lower among women than men across different age and racial/ethnic groups [1-3]. The rates of binge drinking among women have remained stable over the past two decades [4].

It is important to understand the determinants of binge drinking among women because higher rates of binge drinking are linked to increases in alcohol use disorders, injuries and other alcohol-related health problems (including poor health outcomes among babies born to alcohol abusing mothers), psychosocial problems, and high-risk behaviors (e.g., smoking, having multiple sexual partners) [5]. This study examined the rate of binge drinking and factors associated with binge drinking in a representative sample of women in California. We looked at factors in five domains in addition to sociodemographic characteristics: physical health, mental health, help with mental health problems, adverse adult experiences, and adverse childhood experiences. We also examined whether adverse childhood experiences were related to binge drinking as an adult, when adverse experiences and symptoms of poor mental health in adulthood were considered. The purpose was to indicate whether negative childhood experiences may predict binge drinking among adult women above and beyond more recent and current problems. Our goal is to help build binge-drinking prevention and intervention programs that are specific to women by targeting the factors associated with binge drinking.

### Characteristics of women who binge drink

A number of studies have examined sociodemographic factors associated with women's binge drinking. Binge drinking is more common among younger (<30 years) women [6] and binge drinking as a younger woman increases the odds of binge drinking in middle age [7]. In addition, binge drinking is more likely among unmarried and less educated women [5], although studies of highly educated employees found binge drinking rates among women to be high [8,9]. Binge drinking was more common among White and mixed-race than among Hispanic, Black, or Asian women [6]. It is also known to be more common among American Indian women than women in other racial/ethnic groups [10,11]. Rates of binge drinking were higher among non-pregnant than pregnant women [6]. In the domain of mental health, higher rates of post-traumatic stress disorder (PTSD), anxiety, and depression were associated with more binge drinking [5]. In contrast, another study found that women with anxiety and depression had reduced odds of binge drinking [1].

Female victims of violence often engage in self-destructive and maladaptive coping behaviors, including binge drinking. Increased rates of domestic violence among women were associated with increased binge drinking rates [5]. Similarly, women's rates of binge drinking increased from 5.5% among those with no lifetime history of intimate partner violence (IPV), to 12.1% among those with a low level of IPV, to 16.8% among those with a moderate or high level [12]. Binge drinking may occur as a form of self-medication to alleviate symptoms of trauma, anxiety, and depression, and increase feelings of mastery and control [13].

Adverse experiences in childhood also increase the risk for the subsequent problematic use of alcohol such as binge drinking. Childhood sexual abuse was an important predictor of alcohol misuse and binge drinking among adult women [14,15], and adolescent victims of sexual and/or physical assault were more likely than controls to subsequently engage in binge drinking [13]. Each of eight kinds of adverse childhood experiences (verbal, physical, and sexual abuse, had a battered mother, lived with problem drinker/alcoholic or user of street drugs, mental illness in the household, parental separation or divorce, incarcerated household member) was associated with a higher risk of alcohol abuse in a sample of adult women and men [16].

Although adverse childhood experiences have been found to be associated with problematic alcohol use such as binge drinking, studies have not examined whether the childhood events are associated with alcohol misuse even when adult life events and circumstances are also considered. In addition, they have not examined whether poor mental health as an adult mediates the link between adverse childhood experiences and adult binge drinking, as some have hypothesized [17]. Both of these possibilities are important to explore because they have implications for the prevention and treatment of binge drinking. If adverse childhood experiences contribute to binge drinking when concurrent adult functioning is accounted for, and the association of adverse childhood experiences with binge drinking is not fully explained by its impact on poorer adult mental health, then specialized treatment for adverse childhood experiences must be integrated into clinical approaches used in preventing and treating binge drinking.

In summary, in this study we asked the following questions: (1) What is the rate of binge drinking in a representative sample of women in the State of California, (2) What are the sociodemographic, physical and mental health, and adverse adult and childhood experiences associated with binge drinking, (3) Are adverse childhood experiences associated with binge drinking, even when adverse adult experiences and mental health symptoms are already accounted for, and (4) Does poorer adult mental health mediate the association between adverse childhood experiences and adult binge drinking among women.

## Materials and Methods

All procedures for this study were reviewed and approved by Stanford University's Institutional Review Board. We used data from the 2003 and 2004 California Women's Health Survey (CWHS), a population-based, random-digit-dial (not including cell phones) annual probability survey sponsored by the California Department of Health Services and designed in collaboration with several other state agencies and departments. Interviews for the CWHS are conducted in English or Spanish and take approximately 30 minutes to complete.

The response rates for the surveys (i.e., the proportions of eligible households contacted that resulted in a completed interview) were 72% and 74%, respectively. The combined 2-year sample consisted of 6,942 women aged 18 years or older. More detailed information about the sample procedures can be found in [18].

### Measures

#### Binge drinking

Respondents were asked: Considering all types of alcoholic beverages, how many times during the past month did you have five or more drinks on an occasion? Following the CDC definition used prior to 2006, respondents who reported doing so one time or more were classified as binge drinkers; all other respondents, including abstainers, were classified as non-binge drinkers.

#### Sociodemographics

Respondents reported their age, race and ethnicity, marital status, whether children were in the household, education, and employment status. Respondents' reports of household size and annual household income were used to determine whether they were above or below the federal poverty line [19].

#### Physical health

The interview asked: To your knowledge, are you now pregnant (yes/no). It also asked: Do you now smoke cigarettes. Answers were classified as yes (every day or some days) or no (not at all). Respondents' self-reported health was classified as good (i.e., good, very good, or excellent) or fair/poor.

#### Mental health

Symptoms of PTSD were assessed using a five-item screen, demonstrated by Prins et al. [20] to detect current (past 30 days) and clinically significant PTSD symptoms. One item is a general trauma probe in which the interviewer asks if the respondent has ever, in her lifetime, had any experience or experiences that are frightening, horrible or upsetting. Four more items assess the presence or absence of the following PTSD symptoms: intrusive trauma-related thoughts, avoidance of trauma-related cues, emotional numbing, and physiological hyperarousal. Respondents were coded positive for PTSD symptoms if they screened positive for trauma and endorsed ≥3 PTSD items. To assess depression, respondents were asked to report how many of the past 30 days they felt sad, blue, or depressed; and to assess anxiety, they reported how many days they felt worried, tense, or anxious. These items were from the healthy days measure used in the Brief Behavioral Risk Factor Survey [21]. Respondents were coded positive for depression or anxiety if they reported symptoms on ≥14 days of the past 30 days.

#### Mental health help

Poor mental health was assessed by asking respondents to report how many days during the past 30 their mental health (e.g., problems with emotions) was not good. Respondents were coded positive for poor mental health if they reported that their mental health was not good on ≥14 days. In addition, respondents reported whether they had wanted or needed help with personal or family problems from a mental health professional in the past 12 months (yes/no), and whether they had obtained such help (yes/no).

#### Adverse adult experiences

CWHS questions on adverse adult and childhood experiences were from the Traumatic Stress Schedule, a validated self-report measure of traumatic events [22], and subjected to cognitive testing for use in telephone surveys. Respondents reported whether, during the past 12 months, they had experienced intimate partner violence. Specifically, if the respondent answered yes to one or more of 8 items (e.g., partner or former partner had thrown something at her; had pushed, grabbed, shoved, or slapped her), she was classified as having experienced intimate partner violence. Respondents also reported whether they experienced the following adverse experiences during their adulthood (age 18 or after): physical assault (i.e., was beaten up, slapped, punched, kicked, or attacked by a stranger or someone known), sexual assault (i.e., was forced into unwanted sexual activity), or death of a close friend or family member in an accident, homicide, or suicide.

#### Adverse childhood experiences

Respondents reported whether they experienced the following adverse experiences during their childhood (before age 18): was removed from home and placed in foster care; lived with someone who was a problem drinker or alcoholic or used street drugs; lived with someone who was depressed or mentally ill; mother or stepmother was treated violently; lived with someone who went to prison or jail; emotional abuse (i.e., adult in household often or very often swore at, insulted, put down, or made her afraid that she would be physically hurt); physical assault (i.e., was beaten up, slapped, punched, kicked, or attacked by a stranger or someone known); and sexual assault (i.e., was forced into unwanted sexual activity).

### Analyses

The first set of logistic regression analyses examined associations of sociodemographic factors (each entered singly as an independent variable) with binge drinker status. Then, we conducted logistic regression analyses that controlled for the sociodemographic factors found to be significantly associated with binge drinking, and entered one factor (i.e., one variable) in the domain of physical health, mental health, mental health help, adverse adult experiences, or adverse childhood experiences as a predictor. A third set of logistic regressions examined associations of adverse childhood experiences with binge drinking when sociodemographic, adult mental health, and adverse adult experiences that were significantly associated with binge drinking were controlled; the purpose was to determine whether adverse childhood experiences were independently associated with binge drinking after more recent adverse experiences and mental health symptoms as adults were considered. Finally, we conducted a series of regression analyses to determine whether poorer adult mental health mediated between adverse childhood experiences and binge drinking.

## Results

The prevalence of binge drinking was 9.3%. The other sample characteristics are presented in Table 1. We first compared binge drinkers (n = 647) to non-binge drinkers (n = 6,295) on demographics (Table 2). Because binge drinkers were younger and more likely to be unmarried and unemployed, and were somewhat more likely to have an income below the federal poverty line, we controlled for these variables in all subsequent analyses.

**Table 1:** Sample characteristics

Sociodemographics	% of full sample
Age (mean, SD)	32.6, 13.0
Minority/racial group member	50.2
Not married	45.0
No. children in household	49.6
No education beyond high school	37.4
Not employed	45.4
Income below federal poverty line	17.9
Physical health	
Not pregnant	94.7
Smokes	13.6
Fair/poor health	17.3
Mental health	
PTSD symptoms	6.3
Anxiety symptoms	21.8
Depression symptoms	13.7
Overwhelmed by stress	12.5
Mental health help	
Poor mental health	14.2
Perceived need for mental health help	23.2
Utilized mental health help	9.8
Adult victimization	
Intimate partner violence	9.9
Physically assaulted	19.7
Sexually assaulted	10.6
Death of someone close	29.7
Childhood victimization	
Lived in foster care	3.4
Lived with someone abusing substances	22.3
Lived with someone mentally ill	16.8
Lived with mother victimized by violence	18.4
Lived with someone incarcerated	7.6
Emotionally abused by adult in home	18. 4
Physically assaulted	19.1
Sexually assaulted	11.2

**Table 2:** Logistic regressions predicting binge (n = 647) versus non-binge (n = 6295) drinker status from sociodemographics

	Binge drinker			
	
Sociodemographics	*B*	OR	95% CI	*p*
Age (mean, SD)	-.477	.621	.58–.66	<.001
Minority racial/ethnic group member	.084	1.087	.93–1.28	.311
Not married	.980	2.664	2.25–3.16	<.001
No children in household	.135	1.144	.97–1.35	.104
No education beyond high school	.017	1.018	.86–1.20	.838
Not employed	.429	1.535	1.30–1.82	<.001
Income below federal poverty line	.180	1.197	.98–1.46	.081

Note: Each p-value is from a Wald *X*^2 ^(*df *= 1).

Logistic regressions predicting binge drinking from current physical and mental health, and help for mental health problems in the past year, found a significant association for each factor (Table 3). Specifically, binge drinking was associated with not having a current pregnancy, currently smoking, and being in fair or poor rather than good health. In the mental health domain, symptoms of PTSD, anxiety, and depression, as well as feeling overwhelmed by stress, were associated with binge drinking. Consistently, poor mental health, and perceiving the need for and utilizing mental health help in the past year, were associated with binge drinker status. As expected, binge drinking was associated with adverse experiences in adulthood (intimate partner violence, having been physically or sexually assaulted, or having experienced the death of someone close) and in childhood (living with someone abusing substances or mentally ill, or with a mother victimized by violence, and having been physically or sexually assaulted) (Table 4).

**Table 3:** Logistic regressions predicting binge (n = 647) or non-binge (n = 6295) drinker status from physical and mental health and mental health help, controlling for sociodemographics

	Binge drinker			
	
	*B*	OR	95% CI	*p*
Physical Health				
Not pregnant	2.288	9.859	3.40–28.59	<.001
Smokes	.985	2.679	2.21–3.25	<.001
Fair/Poor health	.287	1.333	1.10–1.76	.041
				
Mental health				
PTSD symptoms	.787	2.197	1.69–2.86	<.001
Anxiety symptoms	.478	1.614	1.34–1.94	<.001
Depression symptoms	.497	1.644	1.33–2.04	<.001
Overwhelmed by stress	.613	1.846	1.50–2.28	<.001
				
Mental health help				
Poor mental health	.484	1.622	1.31–2.00	<.001
Perceived need for mental health help, past year	.305	1.357	1.13–1.63	.001
Utilized mental health help, past year	.479	1.614	1.19–2.20	.002

Note: Each p-value is from a Wald *X*^2 ^(*df *= 1).

**Table 4:** Logistic regressions predicting binge (n = 647) or non-binge (n = 6295) drinker status from adult and childhood adverse experiences, controlling for sociodemographics

	Binge drinker			
	
	*B*	OR	95% CI	*p*
Adult adverse experiences				
Intimate partner violence	.542	1.719	1.37–2.15	<.001
Physically assaulted	.670	1.954	1.61–2.36	<.001
Sexually assaulted	.279	1.321	1.02–1.71	.035
Death of someone close	.641	1.898	1.60–2.25	<.001
				
Childhood adverse experiences				
Lived in foster care	.288	1.334	.90–1.99	.157
Lived with someone abusing substances	.480	1.616	1.35–1.94	<.001
Lived with someone mentally ill	.594	1.811	1.50–2.19	<.001
Lived with mother victimized by violence	.587	1.799	1.36–2.39	<.001
Lived with someone incarcerated	.191	1.210	.93–1.58	.161
Emotionally abused by adult in home	.421	1.523	1.26–1.85	<.001
Physically assaulted	.513	1.671	1.39–2.01	<.001
Sexually assaulted	.424	1.527	1.21–1.92	<.001

Note: Each p-value is from a Wald *X*^2 ^(*df *= 1).

Because, as already noted, binge drinking is more common in younger age groups, we examined whether age was a moderator of associations between physical and mental health, mental health help, and adult and childhood adverse experiences and binge drinking. Specifically, we conducted each logistic regression in Tables 3 and 4, adding the interaction of age by the predictor. The interactions of age by poor mental health (*B *= -.272, OR = .762, CI = .63–.72, Wald *X*^2 ^= 9.14, *df *= 1, *p *= .003) and by having lived with someone mentally ill (*B *= -.163, OR-.850, CI = .73–.99, Wald *X*^2 ^= 4.03, *df *= 1, *p *= .045) in predicting binge drinking were significant. There were positive associations of poor mental health and having lived with someone mentally ill with binge drinking in each age group except for the oldest group. That is, among women 65 years old or older only, poor mental health and having lived with someone mentally ill were associated with non-binge drinker status.

We examined whether adverse childhood experiences were still associated with binge drinking when mental health (PTSD, anxiety, depression, overwhelmed) and adverse experiences (intimate partner violence, physical assault, sexual assault, death of someone close) in adulthood were controlled (Table 5). When adulthood factors were also considered, the childhood experiences of having lived with someone who abused substances or who was mentally ill were associated with binge drinking. Specifically, these childhood experiences were associated with roughly double the likelihood of binge drinking. Binge drinking occurred in 14% of women who lived with an individual abusing substances, and in 8% of women who did not. And, binge drinking occurred in 16% of women who lived with an individual who was mentally ill, and in 8% of women who did not.

**Table 5:** Logistic regressions predicting binge (n = 647) or non-binge (n = 6295) drinker status from childhood adverse experiences, controlling for sociodemographics, mental health, and adult adverse experiences

	Binge drinker			
	
Childhood adverse experiences	*B*	OR	95% CI	*p*
Lived in foster care	.044	1.045	.69–1.58	.835
Lived with someone abusing substances	.241	1.273	1.05–1.55	.015
Lived with someone mentally ill	.359	1.431	1.17–1.76	.001
Lived with mother victimized by violence	-.050	.951	.70–1.29	.744
Lived with someone incarcerated	-.121	.886	.67–1.17	.396
Emotionally abused by adult in home	.073	1.075	.87–1.34	.514
Physically assaulted	.188	1.206	.98–1.49	.081
Sexually assaulted	.094	1.099	.85–1.42	.464

Note: Each p-value is from a Wald *X*^2 ^(*df *= 1).

Finally, we examined whether poor mental health as an adult mediated associations between adverse childhood experiences and adult binge drinking. First, we created two new dichotomous variables reflecting adverse childhood experiences: (1) lived with a person who abused substances and/or was mentally ill and/or a mother victimized by violence (50.3%) or not, and (2) was emotionally abused, physically assaulted, or sexually assaulted (33.7%) or not. Regressions showed that living with a disordered/victimized person predicted binge drinking, as did childhood abuse/assault (Table 6).

**Table 6:** Logistic regressions to examine adult mental health as a mediator between childhood adverse experiences and binge drinking.

	Binge drinker			
	
Childhood experiences	*B*	OR	95% CI	*p*
Living with a disordered/victimized person	0.609	1.84	1.25–1.88	<0.001
Abuse/assault	0.578	1.78	1.47–2.06	<0.001
	Adult poor mental health			
Living with a disordered/victimized person	0.964	2.62	2.20–3.13	<0.001
Abuse/assault	1.028	2.80	2.43–3.22	<0.001
	Binge drinker			
Childhood living with a disordered/victimized person	0.380	1.46	1.19–1.80	<.001
Adult poor mental health	0.309	1.36	1.06–1.75	0.016
	Binge drinker			
Childhood abuse/assault	0.500	1.65	1.39–1.96	<0.001
Adult poor mental health	0.357	1.43	1.15–1.78	0.001

Note: Each p-value is from a Wald *X*^2 ^(*df *= 1).

To examine mediation, first, poor mental health (the potential mediator) was the dependent variable in regressions using the new variables as predictors. Living with a disordered or victimized person was associated with poor mental health, as was having been abused or assaulted (Table 6). As already noted (Table 4), poor mental health was associated with binge drinking. A logistic regression predicting binge drinker status from both living with a disordered/victimized person and poor mental health found that both factors retained significant associations with binge drinking (Table 6). Finally, a logistic regression predicting binge drinker status from both childhood abuse/assault and poor mental health also found that both factors retained significant associations (Table 6). That is, adverse childhood experiences represented by living with others' or their own difficult life circumstances retained independent associations with women's binge drinking and was not mediated by poorer mental health status in adulthood.

## Discussion

The CWHS found that the prevalence of binge drinking among adult women in California was 9.3%. This is similar to findings from national surveys of the US population in which 7.3% to 11.8% of women reported drinking five or more drinks on an occasion in the past month [2,23,24].

Compared to women who did not binge drink, binge drinking women were less likely to be pregnant, and more likely to smoke cigarettes and to describe themselves as only in fair or poor health. These results agree with those from other studies that pregnancy is associated with less binge drinking whereas smoking is associated with more binging on alcohol [25,26]. One survey found that 38% of young women reported smoking as the main reason for drinking [27]. Young women who both binge drank and smoked were especially likely to report depressive symptoms [28].

We found that about 13%–14% of the women in this sample had symptoms of depression or overwhelming stress, whereas symptoms of PTSD were less common and symptoms of anxiety were reported by 22% of women. Having symptoms of depression, stress, PTSD, and anxiety were each associated with binge drinking. The association between depression and alcohol misuse has been relatively well-documented in the literature. For example, analyses of the Canadian National Population Health Survey found an association of major depression and binge drinking among women [29]. In a sample of 121 alcohol-dependent women, 31% were diagnosed with depression, and 46% with anxiety, suggesting that women often alleviate stress through drinking [30]. Consistently, we found that women's self-reports of poor mental health, and having perceived the need for and utilized help for mental health problems in the past year, were associated with binge drinking.

As expected, binge drinking was associated with adverse experiences both in adulthood and in childhood. For adult victimization by intimate partner violence or sexual assault in particular, drinking is often a maladaptive means of coping with the traumatic aftermath [31-34]. When victimization by such traumatic events is chronic, binge drinking may be explained by PTSD symptoms [35,36]. That is, alcohol may be used to medicate PTSD-related sleep difficulties, hyperarousal, and other symptoms [37,38].

Victimizing childhood experiences have been consistently linked to problems in childhood that extend into adulthood [39-41]. For example, there was a strong association between adverse childhood experiences such as physical and sexual assault and drug abuse in young women [42]. In addition, longitudinal studies found that mothers' depressive symptoms and history of victimization predicted poorer behavioral outcomes among the children, and that the risk of child behavior problems increased with the number of areas B substance use, mental health, or domestic violence B in which the mother reported difficulties [43,44]. Although the CWHS did not assess respondents' recollections of their psychiatric symptoms in childhood, possibly, those whose mothers had psychiatric difficulties or victimizing experiences had more dysfunction in childhood that then persisted into adulthood. Unfortunately, increased rates of binge drinking associated with childhood and adult victimization may increase the risk for re-victimization [45].

Even after adulthood factors were considered, the childhood experiences of having lived with someone who abused substances (22.3% of the sample did so) or who was mentally ill (16.8% did so) were still associated with binge drinking. Problematic alcohol use is known to be influenced by problematic parental drinking and a family history of alcoholism [46]. While alcoholism has distinct biological and genetic influences [46-48], parental heavy-drinking norms and approval of, or lack of attention to, offspring drinking are also important correlates of risky drinking behaviors and poorer drinking outcomes [49-51]. The risk conferred by living with a dysfunctional adult suggests possible benefits of family-oriented medical and mental health care. Whether providers are initially focused on the adult or the child, there is the potential to help disrupt intergenerational alcohol misuse by considering the entire family or at least the parent-offspring dyad.

Importantly, we found that adverse childhood experiences represented by living in the midst of others' or one's own difficult life circumstances retained independent associations with women's binge drinking and was not mediated by poorer mental health status in adulthood. Thus it may not always be enough to intervene only with binge drinking, or even with the depression and anxiety also associated with binge drinking. Rather, providers working with binge drinkers should consider asking whether adverse childhood experiences have occurred (in this sample, one-half of women lived with an ill or victimized person in their childhood home, and one-third were personally abused or assaulted as children), and then addressing any consequences of such adverse experiences if they have taken place. Research on trauma-focused therapy for women who were victimized in childhood that has shown promise for improving mental health symptoms [52-54] needs to be extended to alcohol and drug outcomes.

## Limitations and Conclusion

The findings must be considered in light of the methods used. The definition of binge drinking was based on a quantitative cut point that did not consider individual factors that affect blood alcohol concentrations (e.g., weight, drinking history, other drugs ingested). Although a clinically-focused definition (e.g., feelings of intoxication) may have produced different results, consuming five or more drinks on a single occasion usually results in intoxication and impairment [55].

Self-reports as used here may somewhat underestimate alcohol consumption compared with diagnostic interviews, but they typically provide more accurate data than laboratory tests and collateral reports [56]. The data on women's health and binge drinking were cross-sectional, and so cause and effect cannot be determined from these results. Because data on adverse experiences as children and adults were retrospective, it is possible that recall bias influenced self-reports. However, previous research suggests that negative experiences in childhood tend to be under- rather than over-reported [57-59], perhaps leading to an underestimation of the strength of associations between childhood and adult hardship and binge drinking. An additional limitation is that the CWHS did not obtain diagnostic information with regard to physical health, PTSD, anxiety, and depression. Although CWHS items were selected from previously conducted national or statewide surveys for comparability, our findings need replication with more sensitive and precise measures of self-perceived physical and mental health.

Identifying characteristics that are commonly found among women who engage in binge drinking is a key step in designing and implementing effective prevention and intervention efforts. Binge drinking prevention programs perhaps should attend to adult mental health symptoms as well as dynamics in the childhood home. Experts working to decrease binge drinking may consider screening for addiction and other psychiatric disorders reported in the childhood home as well as other types of adverse childhood experiences and then addressing the trauma and other consequences of those stressors. Findings that childhood abuse is associated with poor substance use disorder treatment outcomes in adulthood [60] underscore the need to examine comprehensive and integrated approaches to prevent continued binge drinking.

## Competing interests

The authors declare that they have no competing interests.

## Authors' contributions

All the authors contributed to the design of the study and the analysis and interpretation of data, RK and JP helped to design and acquire data from the CWHS, CT managed the literature searches and wrote the first draft of the manuscript, AS managed the analyses. All authors contributed to and approved the final manuscript.
